# Age‐Specific Association Between the Dietary Inflammatory Index and Depression Among Patients With Chronic Kidney Disease

**DOI:** 10.1002/fsn3.71635

**Published:** 2026-03-10

**Authors:** Jialing Zhang, Qing Wang

**Affiliations:** ^1^ Institute of Civil Aviation Aircrew Medical Assessment, Civil Aviation General Hospital Beijing China

**Keywords:** age difference, chronic kidney disease, depression, dietary inflammatory index, mediation

## Abstract

Depression is a common comorbidity in CKD patients. The purpose of this study was to define the age‐specific association between the dietary inflammatory index (DII) and depression among patients with chronic kidney disease (CKD). Data in this cross‐sectional study were collected from the 2005–2018 National Health and Nutritional Examination Survey (NHANES). Weighted multivariable logistic regression models were conducted to assess the relationship between DII and depression in CKD patients across different age groups. A mediation analysis was constructed to clarify the mediating role of inflammation and renal function. A total of 5501 CKD patients were enrolled in this study. A significant association between DII and depression was demonstrated in patients with CKD aged ≤ 70 years, and a linear correlation was uncovered (*p* for nonlinear > 0.05). However, no significant effect was present in patients aged > 70 years. Additionally, residual renal function slightly mediated the role of DII on depression in CKD patients. DII was associated with depressive disorder in CKD patients partially through renal function. Younger CKD patients were more affected, and targeting age‐specific anti‐inflammatory diets could be an intervention strategy to prevent depression in patients with CKD.

## Graphical abstract

Significant association between DII and depression in CKD patients aged ≤ 70 years. Linear association between DII and depression in CKD patients aged ≤ 70 years. Inflammation and residual renal function mediated the association between DII and depression. Age‐specific anti‐inflammatory diets may be a promising intervention for depression in CKD patients.

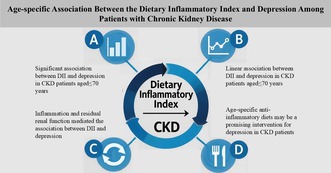

## 1 Introduction

Chronic kidney disease (CKD) is a prevalent global health challenge (GBD Chronic Kidney Disease Collaboration 2020). Depression is a common mental disorder in patients with CKD (Liu et al. 2023), increasing the risk of cognitive dysfunction (Zhou, Zhao, et al. 2024) and mortality (Bautovich et al. 2014). Thus, investigating the specific risk factors for depression can provide insights into better management.

The interplay of lifestyle and inflammatory pathways is believed to contribute to the onset and progression of depression in CKD (Maes et al. 2012; Zhou, Bai, et al. 2024). Notably, extensive research prompted that improving inflammation status played a crucial role in alleviating depression (Guenzani et al. 2019; Lamers et al. 2019). Dietary patterns are well‐recognized for their ability to regulate systemic inflammation levels (Barbaresko et al. 2013). For example, a pro‐inflammatory diet significantly increased the level of white blood cell (WBC) counts, complement component 3, C‐reactive protein (CRP), interleukin 6 (IL‐6), and tumor necrosis factor (TNF)‐α (Phillips et al. 2018), whereas dietary intervention had antioxidant and anti‐inflammatory effects to improve acute kidney injury (Zeng et al. 2023). To quantitatively assess the inflammatory potential of diets, Shivappa, Steck, Hurley, Hussey, and Hébert (2014) designed a literature‐derived, population‐based dietary inflammatory index (DII). Significant positive associations between DII and inflammatory markers were observed, such as IL‐6 and TNF‐α (Shivappa et al. 2015; Tabung et al. 2015). Meanwhile, recent studies have reported a potential mediating role of DII scores in the association between diet and depression among adults (Wang, Fan, et al. 2024; Yang et al. 2024). These findings underscored the potential value of anti‐inflammatory diets in the management of depression.

Previous research has uncovered an indirect association between age and depression (Hosseini et al. 2025). The prevention strategies for depression varied across different age groups. During the COVID‐19 pandemic, depression prevalence was higher in younger adults compared to older adults, and mental health care and economic policies should particularly target younger adults (Collier Villaume et al. 2023). Intergenerational support including emotional support, instrumental support, and financial support could improve health status (Li and Guo 2022). However, different intergenerational support was recommended to individuals aged 45–60, 60–80, and those over 80 to alleviate depressed symptoms (Lu et al. 2025), highlighting an importance to explore the age‐specific effect on depression.

The aforementioned studies indicated that a higher DII was a risk factor for depressive symptoms in the general populations (Akbaraly et al. 2016; Shivappa et al. 2016). However, the association between dietary components and depression in CKD patients and the specific effect of age remains underexplored. This study aimed to explore the age‐specific relationship between DII and depression in CKD patients using data from the National Health and Nutrition Examination Survey (NHANES) from 2005 to 2018.

## 2 Methods

### 2.1 Materials and Methods

NHANES, conducted by the U.S. Centers for Disease Control and Prevention (CDC), is a cross‐sectional design to assess the health and nutritional status of the U.S. population. The study protocol was approved by the National Center for Health Statistics Institutional Review Board, and all participants provided written informed consents.

**FIGURE 1:**
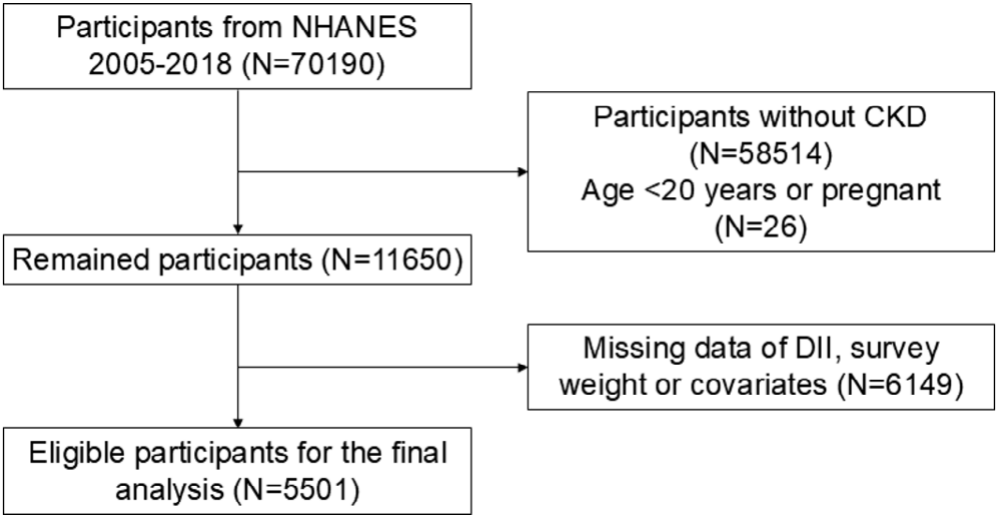
The flowchart of this study.

We analyzed data from NHANES cycles 2005–2018. A total of 11,676 participants with CKD were initially identified from the combined cycles. Exclusion criteria were applied as follows to ensure complete data for the primary exposure (DII), outcome (depression via PHQ‐9), and key covariates: (1) age < 18 years; (2) missing or incomplete dietary recall data necessary for DII calculation; (3) missing survey weights; (4) missing PHQ‐9 for depression assessment; (5) missing key covariates (e.g., age, sex, race/ethnicity, PIR, BMI, hypertension, diabetes, eGFR, etc.). After these exclusions, the final analytic sample comprised 5501 CKD participants. The selection process is illustrated in Figure 1.

### 2.2 Definition of CKD

According to the guideline (Stevens and Levin 2013), CKD is defined based on at least one of the following diagnostic criteria: (1) estimated glomerular filtration rate (eGFR) < 60 mL/min/1.73m^2^; (2) urinary albumin‐to‐creatinine ratio (UACR) ≥ 30 mg/g. The eGFR was calculated by the standardized equation developed by the Chronic Kidney Disease Epidemiology Collaboration (CKD‐EPI) (Levey et al. 2009).

### 2.3 Definition of Depression

Depressive symptoms were measured using the 9‐item Patient Health Questionnaire (PHQ‐9) scale (Kroenke et al. 2001), a widely validated tool for detecting depression (Levis et al. 2019). Each of the nine items is scored on a scale from 0 to 3, yielding a total score ranging from 0 to 27. The PHQ‐9 ≥ 10 was used as the threshold to define depression (Ba et al. 2021; Wang, Fan, et al. 2024).

### 2.4 Definition of DII

NHANES dietary data were collected through two 24‐h dietary recalls. The DII was calculated using 28 nutrients, including energy, carbohydrates, protein, total fat, alcohol, fiber, cholesterol, saturated fat, monounsaturated fatty acids, polyunsaturated fatty acids, omega‐3 fatty acids, omega‐6 fatty acids, niacin, vitamin A, vitamin D, vitamin E, thiamin (vitamin B1), riboflavin (vitamin B2), vitamin B6, vitamin B12, vitamin C, iron, magnesium, zinc, selenium, folic acid, beta‐carotene, and caffeine. The DII calculation follows five steps: (I) assign an inflammation effect score to each nutrient based on prior literature; (II) calculating standardized intake scores (*z*‐scores) = (daily intake of the component—global mean daily intake)/global standard deviation of daily intake; (III) multiply the *z*‐score by the respective inflammatory effect score of each dietary component; (IV) sum the individual DII scores to obtain the overall DII for each participant (Hébert et al. 2019).

### 2.5 Covariates

Baseline demographic characteristics (age, sex, race, education, and poverty income ratio [PIR]) and lifestyle behaviors (smoking status and body mass index [BMI]) were available through the NHANES database. Comorbidities (hypertension, diabetes mellitus, and stroke) were self‐reported by participants. In addition, laboratory indicators (blood urea nitrogen [BUN], creatinine, WBC, and neutrophil) were also collected.

### 2.6 Statistical Analysis

Patients were stratified into three groups based on age (Group 1: ≤ 45 years; Group 2: 45–70 years; Group 3: > 70 years). According to NHANES protocols, second‐day dietary recall weighting was applied to ensure nationally representative estimates. Sample characteristics were presented as weighted mean ± standard deviation for continuous variables and weighted proportions for categorical variables. A weighted logistic regression was used to estimate the association between DII and depression, expressed as odds ratio (OR) and 95% confidence interval (CI). Model 1 was unadjusted, while Model 2 adjusted for age, sex, and race. Model 3 was further adjusted for age, sex, race, BMI, PIR, hypertension, diabetes, stroke, and eGFR. A restricted cubic spline (RCS) model was conducted to investigate potential nonlinear relationships in CKD patients. In addition, mediation analysis was utilized to assess the direct and indirect effects of inflammatory indices and residual renal function on the DII‐depression association. All statistical analysis was performed by R 4.1.2 and Stata software 15.0. A two‐tailed value of *p* < 0.05 was considered statistically significant.

## 3 Results

### 3.1 Baseline Characteristics of Patients

The baseline characteristics of CKD patients stratified by age group were summarized in Table 1. CKD patients with an older age were more likely to be male, had lower educational attainment, and a higher proportion of smoking, hypertension, diabetes, and stroke (*p* < 0.05). Additionally, with increasing age, the level of BMI, serum BUN, creatinine, and PHQ‐9 scores significantly elevated (*p* < 0.05).

**TABLE 1:** Baseline characteristics of study participants stratified by age.

	≤ 45 (*n* = 1070)	45–70 (*n* = 2290)	> 70 (*n* = 2141)	*p*
Age	32.2 ± 0.5	59.4 ± 0.2	77.3 ± 0.1	< 0.001
Sex	399 (37.3)	1148 (50.1)	1077 (50.3)	< 0.001
Race				
Mexican American	228 (21.3)	363 (15.9)	147 (6.9)	< 0.001
Other Hispanic	125 (11.7)	215 (9.4)	105 (4.9)	
Non‐Hispanic White	355 (33.2)	740 (32.3)	1407 (65.7)	
Non‐Hispanic Black	247 (23.1)	785 (34.3)	386 (18.0)	
Other	115 (10.7)	187 (8.2)	96 (4.5)	
Education				
Less than high school	285 (26.6)	702 (30.7)	719 (33.6)	< 0.001
High school	248 (23.2)	536 (23.4)	583 (27.2)	
High school above	537 (50.2)	1052 (45.9)	839 (39.2)	
Poverty income ratio	2.5 ± 0.1	2.9 ± 0.1	2.6 ± 0.1	0.609
BMI	30.4 ± 0.5	31.6 ± 0.3	28.8 ± 0.2	0.001
Smoke	360 (33.6)	1248 (54.5)	1088 (50.8)	< 0.001
Hypertension	288 (26.9)	1563 (68.3)	1568 (73.2)	< 0.001
Diabetes	130 (12.1)	897 (39.2)	651 (30.4)	< 0.001
Stroke	16 (1.5)	198 (8.6)	293 (13.7)	< 0.001
BUN	4.4 ± 0.1	6.1 ± 0.1	7.7 ± 0.1	0.010
Creatinine	79.9 ± 3.2	97.5 ± 1.8	110.0 ± 1.2	< 0.001
eGFR	105.8 ± 1.2	75.4 ± 1.0	55.9 ± 0.6	< 0.001
WBC	7.7 ± 0.1	7.7 ± 0.1	7.6 ± 0.2	0.022
Neutrophil	4.6 ± 0.1	4.7 ± 0.1	4.5 ± 0.1	0.930
DII score	1.5 ± 0.1	1.6 ± 0.1	1.7 ± 0.1	0.406
PHQ‐9 score	3.4 ± 0.2	3.9 ± 0.2	2.9 ± 0.1	0.001

Abbreviations: BMI, body mass index; BUN, blood urea nitrogen; eGFR, estimated glomerular filtration rate; WBC, white blood cell.

### 3.2 Baseline Characteristics of Patients With and Without Depression

The prevalence of depression in CKD patients with different age‐group was observed in Table 2 (≤ 45 years: 11.5%; 45–70 years: 12.3%; > 70 years: 7.3%). Patients with depression in Group 1 (≤ 45 years) and Group 2 (45–70 years) were more likely to be female, had lower educational attainment, and a higher proportion of smoking, hypertension, stroke. In addition, patients who developed depression tended to have lower PIR and higher DII scores (*p* < 0.05). However, the levels of DII in patients aged > 70 years were not significantly different between patients with and without depression.

**TABLE 2:** Baseline characteristics of study participants with and without depression.

	Group 1: ≤ 45			Group 2: 45–70			Group 3: > 70		
	Non‐depression (*n* = 947)	Depression (*n* = 123)	*p*	Non‐depression (*n* = 2946)	Depression (*n* = 414)	*p*	Non‐depression (*n* = 1985)	Depression (*n* = 156)	*p*
Age	35.1 ± 0.9	31.9 ± 1.3	0.240	49.6 ± 0.5	50.7 ± 0.8	0.495	77.4 ± 0.1	77.2 ± 0.4	0.020
Sex	370 (39.1)	29 (23.8)	0.001	1409 (47.8)	138 (33.3)	0.001	1007 (50.7)	70 (44.9)	0.159
Race									
Mexican American	205 (21.6)	23 (18.7)	0.170	517 (17.5)	74 (17.9)	0.087	128 (6.4)	19 (12.2)	< 0.001
Other Hispanic	106 (11.2)	19 (15.4)		290 (9.8)	50 (12.1)		88 (4.4)	17 (10.9)	
Non‐Hispanic White	317 (33.5)	38 (30.9)		945 (32.1)	150 (36.2)		1325 (66.8)	82 (52.6)	
Non‐Hispanic Black	212 (22.4)	35 (28.5)		921 (31.3)	111 (26.8)		357 (18.0)	29 (18.6)	
Other	107 (11.3)	8 (6.5)		273 (9.3)	29 (7.0)		87 (4.4)	9 (5.8)	
Education									
Less than high school	241 (25.4)	44 (35.8)	0.001	829 (28.1)	158 (38.2)	< 0.001	642 (32.3)	77 (49.4)	< 0.001
High school	218 (23.0)	30 (24.4)		688 (23.4)	96 (23.2)		548 (27.6)	35 (22.4)	
High school above	488 (51.5)	49 (39.8)		1429 (48.5)	160 (38.6)		795 (40.1)	44 (28.2)	
Poverty income ratio	1.9 ± 0.2	1.2 ± 0.2	< 0.001	2.8 ± 0.5	1.7 ± 0.1	< 0.001	2.7 ± 0.0	2.1 ± 0.2	< 0.001
BMI	31.8 ± 0.9	30.5 ± 1.4	0.045	30.9 ± 0.2	33.2 ± 0.6	< 0.001	28.5 ± 0.2	30.2 ± 0.8	0.066
Smoke	299 (31.6)	61 (49.6)	0.003	1362 (46.2)	246 (59.4)	< 0.001	997 (50.2)	91 (58.3)	0.145
Hypertension	240 (25.3)	48 (39.0)	0.005	1583 (53.7)	268 (64.7)	< 0.001	1445 (72.8)	123 (78.8)	0.212
Diabetes	112 (11.8)	18 (14.6)	0.597	861 (29.2)	166 (40.1)	< 0.001	582 (29.3)	69 (44.2)	< 0.001
Stroke	11 (1.2)	5 (4.1)	0.020	171 (5.8)	43 (10.4)	0.001	260 (13.1)	33 (21.2)	0.012
BUN	6.6 ± 0.8	7.2 ± 2.7	0.363	5.5 ± 0.1	5.2 ± 0.2	0.936	7.7 ± 0.1	7.0 ± 0.3	0.024
Creatinine	151.3 ± 26.5	171.3 ± 75.3	0.551	91.8 ± 1.8	92.7 ± 3.7	0.752	110.3 ± 1.2	103.5 ± 3.4	0.089
eGFR	85.6 ± 6.1	87.5 ± 6.8	0.525	86.7 ± 0.8	83.9 ± 1.9	0.120	56.0 ± 0.6	59.2 ± 1.7	0.074
WBC	7.7 ± 0.2	8.2 ± 0.8	0.633	7.6 ± 0.7	8.6 ± 0.2	< 0.001	7.5 ± 0.2	7.5 ± 0.2	0.450
Neutrophil	4.6 ± 0.1	4.9 ± 0.5	0.351	4.6 ± 0.1	4.9 ± 0.1	< 0.001	4.5 ± 0.1	4.7 ± 0.2	0.398
DII score	1.5 ± 0.2	2.5 ± 0.4	< 0.001	1.6 ± 0.1	2.6 ± 0.2	< 0.001	1.7 ± 0.1	1.6 ± 0.2	0.069
PHQ‐9 score	2.9 ± 0.4	13.0 ± 0.7	< 0.001	2.4 ± 0.1	14.3 ± 0.3	< 0.001	2.2 ± 0.1	13.8 ± 0.4	< 0.001

Abbreviations: BMI, body mass index; BUN, blood urea nitrogen; eGFR, estimated glomerular filtration rate; WBC, white blood cell.

### 3.3 Relationship Between DII and Depression in CKD Patients Across Different Age‐Group

In the overall population, a higher DII was significantly associated with depression in CKD patients (Model 1: OR 1.14, 95% CI 1.03, 1.27). After adjusting for potential confounding, the correlation between DII score and depression was slightly attenuated (Model 3: OR 1.04, 95% CI 1.00, 1.10) (shown in Table 3).

**TABLE 3:** Association between DII score and depression in CKD patients.

	OR (95% CI), *p*		
	Model 1	Model 2	Model 3
Total			
DII score (per 1‐unit increase)	1.14 (1.03, 1.27), *p* = 0.013	1.13 (1.05, 1.21), *p* = 0.019	1.04 (1.00, 1.10), *p* = 0.050
DII score (groups)			
Tertile 1	Ref.	Ref.	Ref.
Tertile 2	1.20 (0.79, 1.82), *p* = 0.431	1.18 (0.96, 1.46), *p* = 0.073	0.97 (0.72, 1.30), *p* = 0.718
Tertile 3	1.54 (1.04, 2.27), *p* = 0.023	1.46 (1.30, 1.64), *p* = 0.005	1.06 (0.87, 1.30), *p* = 0.326
≤ 45			
DII score (per 1‐unit increase)	1.22 (1.09, 1.37), *p* = 0.001	1.19 (1.01, 1.34), *p* = 0.003	1.14 (1.00, 1.30), *P* = 0.050
DII score (groups)			
Tertile 1	Ref.	Ref.	Ref.
Tertile 2	1.44 (0.85, 2.43), *p* = 0.179	1.33 (0.78, 2.26), *p* = 0.298	1.38 (0.77, 2.45), *p* = 0.275
Tertile 3	1.98 (1.21, 3.25), *p* = 0.007	1.78 (1.08, 2.93), *p* = 0.024	1.84 (1.06, 3.19), *p* = 0.030
45–70			
DII score (per 1‐unit increase)	1.18 (1.11, 1.25), *p* < 0.001	1.15 (1.08, 1.22), *p* < 0.001	1.10 (1.03, 1.18), *p* = 0.004
DII score (groups)			
Tertile 1	Ref.	Ref.	Ref.
Tertile 2	1.30 (0.98, 1.72), *p* = 0.065	1.22 (0.92, 1.61), *p* = 0.174	1.20 (0.90, 1.60), *p* = 0.221
Tertile 3	1.74 (1.34, 2.27), *p* = 0.001	1.55 (1.18, 2.03), *p* = 0.001	1.52 (1.14, 2.01), *p* = 0.004
> 70			
DII score (per 1‐unit increase)	1.08 (0.98, 1.19), *p* = 0.104	1.07 (0.97, 1.18), *p* = 0.152	0.99 (0.89, 1.10), *p* = 0.836
DII score (groups)			
Tertile 1	Ref.	Ref.	Ref.
Tertile 2	1.03 (0.67, 1.57), *p* = 0.890	1.02 (0.67, 1.56), *p* = 0.927	0.85 (0.54, 1.35), *p* = 0.503
Tertile 3	1.33 (0.89, 1.98), *p* = 0.159	1.29 (0.86, 1.93), *p* = 0.212	0.99 (0.64, 1.54), *p* = 0.967

*Note:* Model 1: unadjusted; Model 2: adjusted for age, sex, race; Model 3: adjusted for age, sex, race, PIR, BMI, hypertension, diabetes, stroke and eGFR.

Notably, the association between DII and depression varied across different age‐group. In the Group 1 (≤ 45 years), increase of DII independently correlated with higher odds of depression in patients with CKD after adjusting for other covariates (Model 3: OR 1.14, 95% CI 1.00, 1.30). And the highest depression risk was observed in the highest tertile of DII (Model 3: OR 1.84, 95% CI 1.06, 3.19). Additionally, a similar trend was presented in patients with CKD in the Group 2 (45–70 years). However, no significant association between DII and depression was observed in the Group 3 (> 70 years).

### 3.4 Nonlinear Relationships of DII With Depression

**FIGURE 2:**
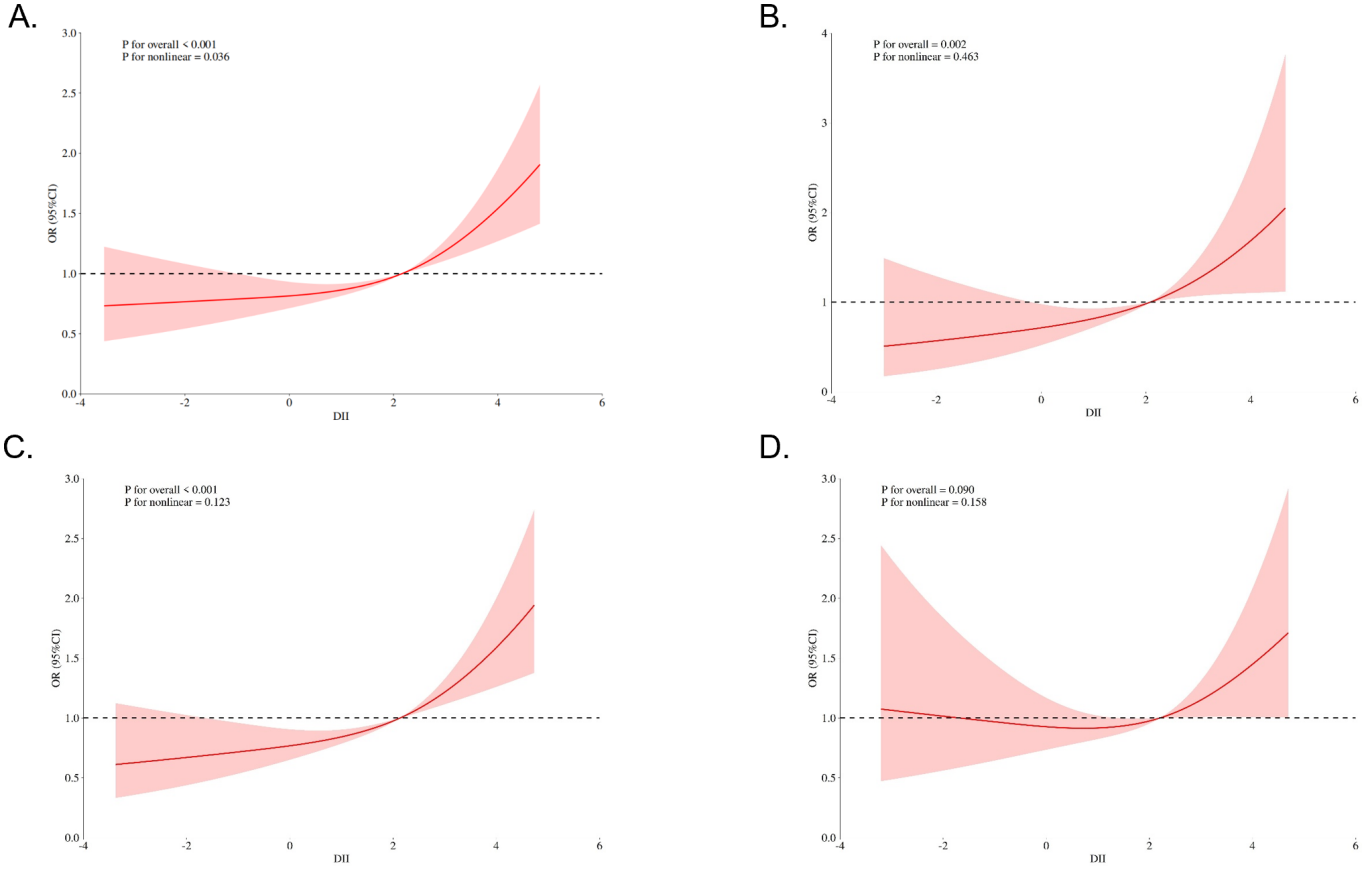
RCS curve for the relationship between DII and the risk of depression (A. Total; B. Age ≤ 45 years; C. Age 45–70 years; D. Age > 70 years).

RCS analysis yielded a non‐linear relationship between DII score and depression in the overall patients with CKD (shown in Figure 2A). In patients aged ≤ 45 years and aged 45–70 years (shown in Figure 2B,C), DII displayed a significant linear relationship with the depression risk (*p* for nonlinear > 0.05). However, DII revealed nonsignificant relationship with the depression risk in patients aged > 70 years (*p* for overall > 0.05) (shown in Figure 2D).

### 3.5 Mediation Analysis

**FIGURE 3:**
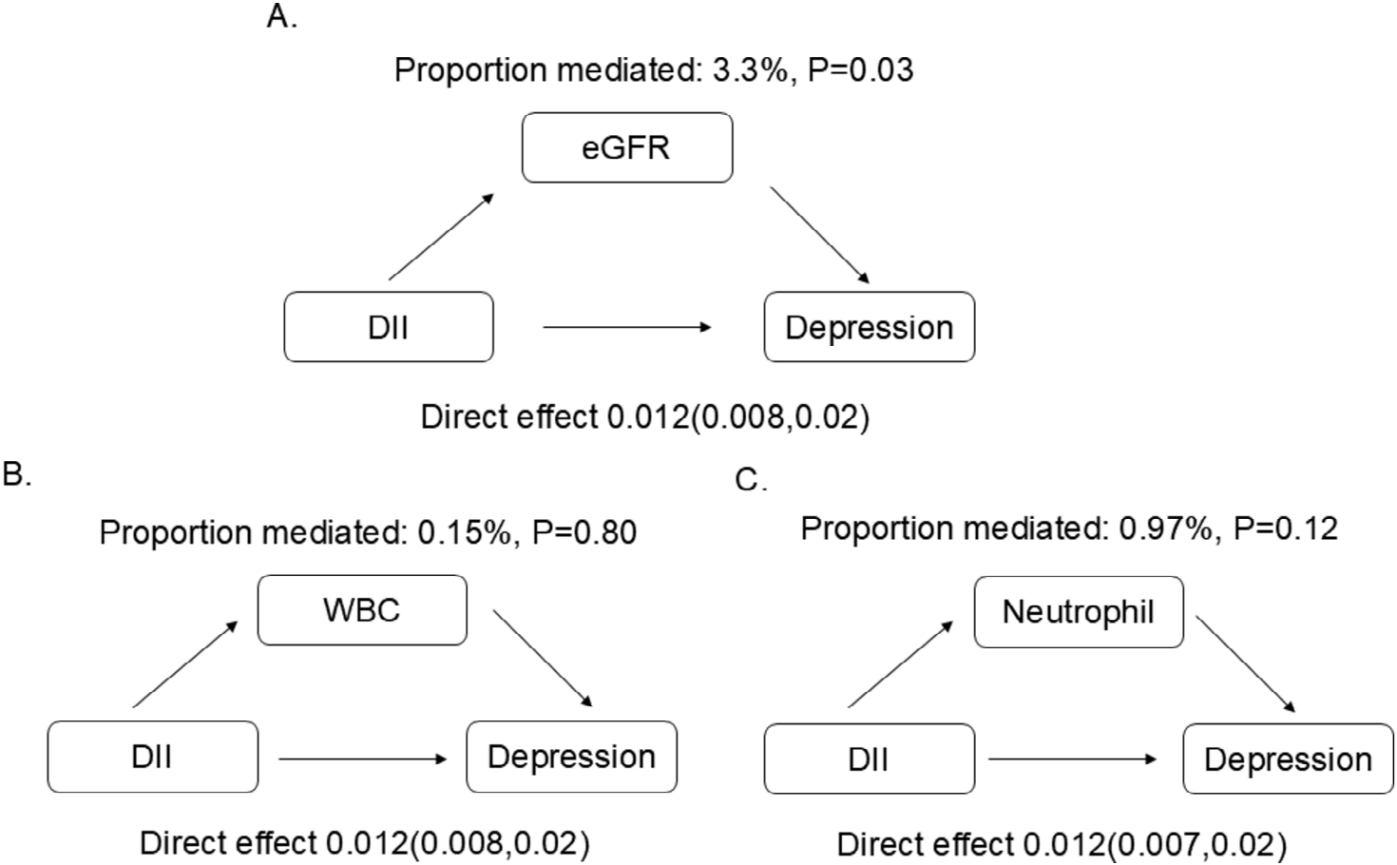
Mediation analyses (A: eGFR; B: WBC; C: Neutrophil). (A) Significant association between DII and depression in CKD patients aged ≤ 70 years. (B) Linear association between DII and depression in CKD patients aged ≤ 70 years. (C) Inflammation and residual renal function mediated the association between DII and depression. (D) Age‐specific anti‐inflammatory diets may be a promising intervention for depression in CKD patients.

Given the potential effect of inflammation and renal function on depression (Herder et al. 2025; Ko et al. 2025), we conducted mediation analyses to explore the indirect effects of eGFR, WBC, and neutrophil in the DII‐depression association. Weighted multivariate linear regression analysis (shown in Table 4) indicated positive associations between DII and WBC/neutrophil counts, and a negative association between DII and eGFR. Hence, eGFR, WBC, and neutrophil were used as mediators in the subsequent mediation analysis. Figure 3 demonstrated that eGFR partially mediated the association of the DII score and depression in CKD populations, highlighting the role of renal function in this relationship.

**TABLE 4:** Multiple linear regression between DII score and related factors.

	*β* (95% CI), *p*
eGFR	−0.009 (−0.013, −0.005), *p* < 0.001
WBC	0.008 (0.006, 0.016), *p* = 0.035
Neutrophil	0.033 (0.001, 0.066), *p* = 0.041

*Note:* Adjusted for age, sex, race, PIR, BMI, hypertension, diabetes, and stroke.

## 4 Discussion

In this study, we uncovered an age‐specific association between higher DII and depression risk in CKD patients. The prognostic value of DII was more pronounced in CKD patients aged ≤ 70 years, with evidence of a linear relationship. Additionally, eGFR slightly mediated the effect of DII on depression, suggesting a potential interplay between diet, renal function, and mental health in CKD populations. Our findings emphasized the need for tailored dietary management across different age groups to prevent depression in patients with CKD.

The DII is a validated, literature‐derived tool designed to quantify the overall inflammatory potential of an individual's diet (Shivappa, Steck, Hurley, Hussey, and Hébert 2014). It has been identified as an independent risk factor for several CKD‐related complications, including hyperparathyroidism (Qin et al. 2021), sarcopenia (Mansouri et al. 2024), and osteoporosis (Meng et al. 2023). Conversely, diets with lower DII scores, such as Mediterranean‐style patterns, have been linked to improved renal function parameters (Marrone et al. 2025), reduced risk of cognitive impairment (Perez et al. 2023), and potentially decreased cardiovascular risk (Rovira et al. 2024). These observations supported the hypothesis that anti‐inflammatory dietary interventions may represent a promising, multifaceted approach to CKD management.

A robust body of evidence implicated chronic inflammation in the pathogenesis of depression among patients with CKD. Depressed CKD patients exhibited higher levels of pro‐inflammatory cytokines (Jayakumar et al. 2023) and dietary inflammation potential in comparison to the general population (W. Chen et al. 2021). Consistent associations between higher DII scores and increased risk of depressive symptoms have also been reported across diverse populations (Luo et al. 2023; Zhang, Wu, et al. 2022). Moreover, DII has been shown to mediate the relationship between lifestyle patterns and depression in patients with diabetes (Wang, Fan, et al. 2024) and multiple sclerosis (Saul et al. 2024), suggesting its broader role in mental health regulation. Notably, age showed a significant interaction with lifestyles, social factors and environmental factors on depressed symptoms (Anderson et al. 2024; Ji and Wang 2024; Lu et al. 2025). However, the age‐specific association between DII and depression in CKD patients remains uncertain. To address this research gap, the present study particularly focused on the role of age in modulating the DII‐depression association among patients with CKD.

Our study provided novel evidence of an age‐specific association between higher DII scores and increased prevalence of depression in CKD patients, particularly in patients aged ≤ 70 years. Several interconnected mechanisms may underlie the link between diet‐driven inflammation and depression, particularly in the context of CKD. Pro‐inflammatory diets, as quantified by elevated DII, robustly promoted systemic inflammation characterized by increased circulating levels of CRP, IL‐6, and TNF‐α (Shivappa, Steck, Hurley, Hussey, Ma, et al. 2014). This systemic inflammatory milieu could activate microglia, trigger neuroinflammation in mood‐regulating brain regions, and impair neurotransmitter metabolism (Hoogland et al. 2018). For example, saturated fat‐rich diets may induce neuroinflammation via toll‐like receptor‐4 pathway and subsequent NF‐κB activation (Kang et al. 2016), whereas fiber and bioactive compounds in garlic exerted opposing effects by suppressing NLRP3 inflammasome activity and cytokine release (Arreola et al. 2015; Spano et al. 2022). The gut–brain axis represented another pivotal pathway (Li et al. 2025). Pro‐inflammatory dietary patterns disrupted microbial composition, favoring pro‐inflammatory taxa, reducing butyrate‐producing genera, and promoting gut‐derived uremic toxin production (e.g., indoxyl sulfate, p‐cresyl sulfate) (Gao et al. 2023; Merino Del Portillo et al. 2024). Furthermore, Western‐style dietary patterns have been linked to endothelial dysfunction and atherogenic endothelial activation (Defagó et al. 2014), which may compromise cerebral perfusion and thereby predispose individuals to neuropsychiatric sequelae. In CKD, these effects are markedly amplified by disease‐specific factors, including uremia‐induced dysbiosis, reduced gut barrier integrity, and impaired renal clearance of gut‐derived toxins (Wagner et al. 2025). Accumulated uremic toxins can directly compromise blood–brain barrier integrity, exacerbate neuroinflammation, disrupt serotonin and kynurenine pathways, and intensify oxidative stress—collectively contributing to depressive symptomatology (Bobot et al. 2025; Hassamal 2023).

Our mediation analysis supported this conceptual framework, revealing that residual renal function (eGFR) partially mediated the association between DII and depression in CKD patients. This finding suggested that dietary inflammation may indirectly exacerbate depressive symptoms by accelerating renal deterioration. The likely sequence involved diet‐induced systemic inflammation and oxidative stress promoting CKD progression and eGFR reduction (Rouhani et al. 2019; Xu et al. 2024). Diminished renal clearance then fostered accumulation of gut‐derived uremic toxins, which in turn exacerbated neuroinflammation, increased blood–brain barrier permeability, and ultimately heightened depressive risk (Bobot et al. 2025). This pathway aligned with the observed dose‐dependent relationship between eGFR decline and incident depression (Ko et al. 2025), and with prior evidence of renal function mediating DII‐related outcomes in older populations (Li et al. 2024). Although the mediated proportion is modest, it carried potential clinical relevance in the context of CKD‐associated depression. Interventions preserving eGFR, such as anti‐inflammatory dietary regimens, could confer cumulative mental health benefits over time. Conversely, other evidence indicated the relative stability of depressive symptoms despite eGFR changes (Knapp et al. 2024), highlighting the multifactorial etiology of depression in CKD and the need to prioritize direct anti‐inflammatory strategies.

Prior studies have implicated inflammation as a key mediator linking dietary patterns—such as fiber intake—to depressive symptoms (Chen et al. 2022; Zhang, Cui, et al. 2022), with inflammatory markers also mediating associations between DII and depression (Y. Chen et al. 2022). However, our analysis in CKD patients found no significant mediating role for circulating leukocyte counts in the DII‐depression association. These findings highlighted the potential of anti‐inflammatory dietary interventions to directly benefit mental health in CKD, independent of classical circulating inflammatory pathways. Supporting evidence indicated that dietary interventions could effectively reduce uremic toxins, lower pro‐inflammatory cytokines (Wathanavasin et al. 2025), improve inflammation profiles (Hassan et al. 2023; Wang, Yin, et al. 2024), and slow CKD progression (Pan and Feng 2025). By attenuating systemic and neuroinflammation and preserving residual renal function, these strategies may offer a dual mechanism for mitigating depressive burden. Nevertheless, direct interventional evidence evaluating anti‐inflammatory dietary specifically for depression outcomes in CKD patients remains limited (Lee et al. 2023). Well‐designed prospective randomized controlled trials are urgently needed to assess whether Mediterranean‐style, high‐fiber, or low‐DII dietary patterns can effectively prevent or alleviate depressive symptoms in CKD patients.

Consistent associations between anti‐inflammatory diets and reduced depressive symptoms have been documented in older adults (Lv et al. 2022; Xiao and Huang 2022). Importantly, we found an age‐specific association between higher DII scores and depression risk in CKD patients, with the association strongest in those aged ≤ 70 years‐ aligning with previous research (Huang and Zhu 2025). Although depression is widely common in elderly people (Andreas et al. 2017), we observed a lower prevalence of depression in CKD patients > 70 years compared to younger groups, a pattern echoed in other chronic disease cohorts (Niraula et al. 2024). This may reflect a true lower burden in the oldest‐old (e.g., survivor bias or altered symptom expression), under‐detection due to somatic symptom overlaps with aging/CKD comorbidities, or reduced willingness to report in very elderly individuals. These factors may have jointly reduced the ability to detect diet‐depression associations in the oldest group.

Several mechanisms may underlie the observed age‐specific pattern. First, individuals ≤ 70 years were more likely to consume pro‐inflammatory Western dietary patterns with greater dietary diversity exposure, thereby amplifying observable DII effects (Qin et al. 2025; Reininger et al. 2017). In contrast, very elderly patients often exhibited marked dietary monotony, reducing DII variability and attenuating detectable associations (Lee 2025). Second, age‐related differences in inflammatory response, coping style, and symptom expression may modify the diet‐depression link. Older adults typically exhibited more proactive coping and lower emotional reactivity (Neubauer et al. 2019), potentially buffering inflammatory effects on depression. Third, older adults with CKD were frequently accompanied by decreased physical activity, sarcopenia, diminished nutritional quality, and altered nutrient absorption/metabolism (Ashton et al. 2020; Russ et al. 2020), all of which further constrain dietary diversity and mask pro‐inflammatory diet effects. Additionally, while hormonal (e.g., post‐menopausal changes) and circadian rhythm alterations have been implicated in age‐depression interactions (Hosseini et al. 2025), their specific contribution to DII‐related pathways in CKD remains uncertain and requires further investigation. Taken together, these age‐specific physiological, behavioral, and dietary differences suggested that anti‐inflammatory dietary interventions may yield the greatest benefit for preventing or mitigating depression in CKD patients aged ≤ 70 years. For those > 70 years, nutritional strategies should instead emphasize overall diet quality, muscle preservation, and multimorbidity management to support mental health.

Although our study demonstrated a significant, age‐specific association between higher DII scores and depression risk in CKD patients aged ≤ 70 years, it would be premature to advocate DII as a standalone predictor or routine screening tool for depression in clinical practice. Additionally, implementing anti‐inflammatory diets may face substantial barriers in socioeconomically disadvantaged CKD populations, including food insecurity, higher costs of nutrient‐dense foods, and limited access, potentially exacerbating health disparities and reducing adherence. To improve accessibility, recommendations should prioritize affordable alternatives alongside multidisciplinary support, and policy interventions. Several limitations should also be acknowledged. First, the cross‐sectional design of NHANES precludes assessment of causality or temporality between DII and depression; reverse causation (e.g., depressed individuals choosing more pro‐inflammatory foods) cannot be ruled out. Prospective longitudinal studies are essential to clarify whether elevated DII precedes incident depression or worsening depressive symptoms in CKD. Second, dietary recall data provided only a partial snapshot of habitual dietary intake and could not accurately reflect long‐term dietary patterns, potentially introducing recall bias. Third, despite adjustment for a comprehensive set of confounders, residual confounding from unmeasured or imperfectly measured factors remains possible. Fourth, although the overall sample size was reasonable, the number of depressed cases in the > 70 years subgroup was limited, reducing statistical power to detect potentially modest associations in the oldest age stratum. Fifth, while our results implied potential benefits from anti‐inflammatory dietary interventions, direct interventional evidence—particularly randomized controlled trials targeting depression outcomes in this population—is scarce. This constrains our ability to quantify efficacy, safety, or long‐term adherence of such interventions. These limitations highlighted the need for future research, including multicenter prospective cohort studies with repeated dietary and mental health assessments, as well‐powered randomized trials evaluating age‐stratified anti‐inflammatory dietary interventions. Mechanistic investigations elucidating the specific pathways linking dietary inflammation to depression in CKD also remain critically underdeveloped and warrant priority.

## 5 Conclusion

In conclusion, this cross‐sectional study demonstrated a significant age‐specific association between higher DII scores and depression in CKD patients, characterized by a linear relationship that is most pronounced in individuals aged ≤ 70 years, with partial mediation through reduced eGFR. These results positioned pro‐inflammatory dietary patterns as a potentially modifiable risk factor for depression in younger and middle‐aged CKD populations. Prospective longitudinal studies and randomized controlled trials are now required to establish causality, validate DII as a screening biomarker, and evaluate the efficacy of targeted anti‐inflammatory dietary interventions.

## Author Contributions

Q.W. contributed to the study concept and design; J.Z. and Q.W. contributed to data collection; J.Z. contributed to the statistical analysis; J.Z. and Q.W. contributed to the original draft. All authors approved the final version of the manuscript.

## Funding

The authors have nothing to report.

## Ethics Statement

All participants submitted written informed consent and were approved by the NCHS Research Ethics Review Board.

## Consent

The authors have nothing to report.

## Conflicts of Interest

The authors declare no conflicts of interest.

## Data Availability

The data that support the findings of this study are available from the corresponding author upon reasonable request.
